# Phase Behavior of Melts of Diblock-Copolymers with One Charged Block

**DOI:** 10.3390/polym11061027

**Published:** 2019-06-10

**Authors:** Alexey A. Gavrilov, Alexander V. Chertovich, Igor I. Potemkin

**Affiliations:** 1 Physics Department, Lomonosov Moscow State University, 119991 Moscow, Russia; chertov@polly.phys.msu.ru; 2 Semenov Institute of Chemical Physics, 119991 Moscow, Russia; 3 DWI-Leibniz Institute for Interactive Materials, 52056 Aachen, Germany

**Keywords:** microphase separation, polyelectrolytes, poly(ionic liquids), dissipative particle dynamics

## Abstract

In this work, we investigated the phase behavior of melts of block-copolymers with one charged block by means of dissipative particle dynamics with explicit electrostatic interactions. We assumed that all the Flory–Huggins χ parameters were equal to 0. We showed that the charge- correlation attraction solely can cause microphase separation with a long-range order; a phase diagram was constructed by varying the volume fraction of the uncharged block and the electrostatic interaction parameter λ (dimensionless Bjerrum length). The obtained phase diagram was compared to the phase diagram of “equivalent” neutral diblock-copolymers with the non-zero χ-parameter between the beads of different blocks. The neutral copolymers were constructed by grafting the counterions to the corresponding co-ions of the charged block with further switching off the electrostatic interactions. Surprisingly, the differences between these phase diagrams are rather subtle; the same phases in the same order are observed, and the positions of the order-disorder transition ODT points are similar if the λ-parameter is considered as an “effective” χ-parameter. Next, we studied the position of the ODT for lamellar structure depending on the chain length *N*. It turned out that while for the uncharged diblock copolymer the product χ_cr_*N* was almost independent of *N*, for the diblock copolymers with one charged block we observed a significant increase in λ_cr_*N* upon increasing *N*. This can be attributed to the fact that the counterion entropy prevents the formation of ordered structures, and its influence is more pronounced for longer chains since they undergo the transition to ordered structures at smaller values of λ, when the electrostatic energy becomes comparable to *k_b_T*. This was supported by studying the ODT in diblock-copolymers with charged blocks and counterions cross-linked to the charged monomer units. The ODT for such systems was observed at significantly lower values of λ, with the difference being more pronounced at longer chain lengths *N*. The fact that the microphase separation is observed even at zero Flory–Huggins parameter can be used for the creation of “high-χ” copolymers: The incorporation of charged groups (for example, ionic liquids) can significantly increase the segregation strength. The diffusion of counterions in the obtained ordered structures was studied and compared to the case of a system with the same number of charged groups but a homogeneous structure; the diffusion coefficient along the lamellar plane was found to be higher than in any direction in the homogeneous structure.

## 1. Introduction

Microphase separated polymer systems have attracted great attention due to their wide range of possible applications [1]. Despite the significant success in studying the phase behavior of uncharged copolymers, in which immiscible blocks tend to segregate, while their connectivity into the chains leads to the microphase separation instead of the macrophase separation [2,3,4,5,6,7,8,9,10], there is no comprehensive description of the problem of microphase separation in polyelectrolytes (polymers containing charged groups). The presence of charged groups and, as a result, the effects related to that (such as correlation attraction, ion association processes, counterion entropy etc.) significantly alters the system behavior. The investigation of the microphase separation in polyelectrolytes is of topical interest because nanostructured ion-conducting membranes have promising applications in a variety of energy storage and conversion devices, such as lithium-ion batteries, fuel cells, and others [11,12,13]. Moreover, there are other possible applications for such nanostructures systems in nanoelectronics, nanophotonics, and as membranes for separation etc. Microphase separated nanostructured systems have a number of advantages compared to homogeneous ones: Different phases can have different properties, for instance, one can make the material mechanically stable and tough, while the other can have good ion conductivity. Therefore, understanding the principles of the formation of phases with different topologies in polyelectolytes will allow the creation of new materials with significantly improved properties for the aforementioned applications.

During the last years the problem of microphase separation in polyelectrolytes has attracted the increasing attention of researchers. In the majority of work, such systems are studied experimentally. As a good example, one can refer to the review [14] in which the influence of electrostatic interactions on the phase behavior of copolymers with charged and neutral blocks is discussed. In particular, it was shown [15] that for sulfonated poly(styrene)-*b*-poly(methylbutylene) diblock-copolymer the degree of sulfonation of the charged block plays an important role in determining the system morphology. Varying the sulfonation degree between 0 and 44.7%, the morphologies were found to change from disordered to gyroid to lamellae to hexagonally perforated lamellae (HPL). However, it was noted that no morphologies other than the ones present on the phase diagram of uncharged diblock-copolymer (for example, see Reference [8]) were found. An “unconventional” morphology was found in Reference [16]: Inverted cylinders were observed for sulfonated poly-(styrene)-b-poly(isoprene) with the sulfonated polystyrene minor component forming the matrix. The authors attributed such behavior to the counterions entropy; however, the inverted cylinders were formed only immediately after the film formation, and thermal annealing destroyed the long-range order. It is apparent that there is a need for a significant amount of additional investigation in order to understand the physical principles of the formation of microphase separation in such systems [14].

One of the most interesting and dynamically developing areas of polyelectrolytes studies is the investigation of the properties of poly(ionic liquids). In short, polyionic liquids are a special type of polyelectrolyte which carry an ionic liquid IL species in each of the repeating units [17]. Such architecture allows one to combine the advantages of polymers and ionic liquids in one material. For instance, “classical” polymer electrolytes could not play the role of solid ion conductors: Due to the extensive ion-pairing, only materials with very low bulk conductivity are usually obtained in absence of a polar solvent. A good example of such behavior is the well-known material Nafion, which conducts only when being swollen with a significant amount of water. This problem is not present for poly (ionic liquids), as they demonstrate high ionic conductivity even in the “dry” state (without a “conventional” solvent), which significantly improves the mechanical properties of such conducting materials. Moreover, there are a lot of types of anions and cations which can be used, and the resulting polymer material can conduct either cations or anions depending on which ion is linked to the polymer. All these circumstances allow one to fine tune the properties of the system. One of the most comprehensive investigations of the phase behavior of block copolymers with one block being a poly(ionic liquid) is Reference [18]. In that work, perfectly ordered structures of various types were obtained for different fractions of the polyelectrolyte block, including lamellar, cylindrical, and spherical. In Reference [19], the microphase separated systems formed by diblock-copolymers with poly(ionic liquid) block were shown to have a higher conductivity compared to the ones with weak separation; the systems that tend to form bicontinuous structures demonstrated the best conductivity among all others. The influence of the presence of microphase separation on conductivity was also studied in Reference [20]; it was found that hydroxide conductivity is higher in microphase-separated diblock-copolymers with one charged block than in analogous conducting homopolymer (and much higher compared to random copolymers comprised of the same monomers), which was explained by the presence of conducting channels in the microphase-separated systems. More complex chain architectures are also being studied: In a recent paper [21] the combination of properties of triblock copolymers with charged side blocks was found to be better than that described in the literature for other types of electrolytes.

As for the theoretical and simulation work, there is still no more or less systematic description of the microphase separation in polyelectrolytes, which is especially apparent if one considers the phase behavior of neutral copolymers that is studied very well even for different polymer chain architectures. Moreover, due to the presence of several types of interactions and length scales, it is a complicated task to create a consistent theoretical description of such systems. One of the most recent and complete investigation of the microphsase separation in melts of diblock-copolymers with one charged block was carried out in the papers [22,23]. In those works, a combination of the self-consistent field theory and the liquid state theory accounting for the local charge organization was used; the latter play an important, if not the main, role in the self-organization of polyelectrolytes. It was shown that the presence of charged monomer units and differences in the dielectric permittivity between phases formed by different blocks significantly change the phase diagram, making it asymmetrical, and allowing one to obtain ordered structures even at a zero Flory–Huggins parameter. Furthermore, it was shown that the region of ordered structures dramatically shrinks upon increasing the fraction of charged units higher than 15%, which is rather interesting: It seems that an increase in the fraction of charged units should facilitate the microphase separation because of the intensification of the charge correlation attraction. Therefore, there is a need for a detailed investigation of the system with particle methods, in which the correlation attraction as well as fluctuations (which play in important role in the microphase separation phenomenon) are taken into account automatically.

In this work, we addressed the question of microphase separation in melts of diblock-copolymers with one charged block. We focused on the effect of the presence of charged groups and mobile counterions and therefore investigated the case of completely compatible blocks (Flory–Huggins parameter χ = 0). The behavior of such systems was compared to that of corresponding uncharged diblock-copolymers as well as copolymers with counterions grafted to the corresponding co-ions of the charged block. Finally, we studied the influence of the ordering in the system on the counterion diffusion.

## 2. Model and Methods

In this work we used dissipative particle dynamics with explicit electrostatic interactions as the simulation method. First, we give a brief description of the standard model without electrostatic interactions. Dissipative particle dynamics (DPD) is a version of the coarse-grained molecular dynamics adapted to polymers and mapped onto the classical lattice Flory–Huggins theory [24,25,26,27]. It is a well-known method which has been used to simulate properties of a wide range of polymeric systems, such as single chains in solutions, microphase separation in polymer melts and networks. In short, macromolecules are represented in terms of the bead-and-spring model (each coarse-grained bead usually represents a group of atoms), with beads interacting by a conservative force (repulsion) $F_{ij}^{c}$, a bond stretching force (only for connected beads)$\;F_{ij}^{b}$, a dissipative force (friction)$\;F_{ij}^{d}$, and a random force (heat generator) $\;F_{ij}^{r}$. The total force is given by:

(1) $$F_{i}=\underset{i\neq j}{\sum }\left(F_{ij}^{c}+F_{ij}^{b}+F_{ij}^{d}+F_{ij}^{r}\right).$$

The soft-core repulsion between *i*- and *j*-th beads is equal to:

(2) $$F_{ij}^{c}=\{\begin{array}{c} a_{\alpha \beta }(1-r_{ij}/R_{c})r_{ij}/r_{ij},\;r_{ij}\leq R_{c}\\ 0,r_{ij}>R_{c} \end{array},$$

where ***r_ij_*** is the vector between *i*-th and *j*-th bead, *a_αβ_* is the repulsion parameter if the particle *i* has the type *α* and the particle *j* has the type *β* and *R_c_* is the cutoff distance. *R_c_* is basically a free parameter depending on the volume of real atoms each bead represents [27]; *R_c_* is usually taken as the length scale, i.e., *R_c_* = 1.

If two beads (*i* and *j*) are connected by a bond, there is also a simple spring force acting on them:

(3) $$F_{ij}^{b}=-K(r_{ij}-l_{0})\frac{r_{ij}}{r_{ij}},$$

where *K* is the bond stiffness and *l*_0_ is the equilibrium bond length.

Here, we do not give a more detailed description of the standard DPD model (without electrostatic interactions); it can be found elsewhere [27].

To take into account the electrostatic interactions, we use the method described in Reference [28]. The main problem in studying electrostatic interactions in DPD is that, due to the soft-core nature of the repulsion force, oppositely charged particles would overlap if the unmodified Coulomb potential was used; the modifications described in Reference [28] is one of the approaches to solving this problem. The electrostatic force between two charged beads is calculated using the following expression:

(4) $$F_{ij}^{e}=\frac{q_{i}q_{j}}{4\pi \varepsilon \varepsilon_{0}}\left\{ \begin{array}{l} \frac{r_{ij}}{r_{ij}^{3}}\sin^{6}\left(\frac{2\pi r_{ij}}{4D}\right),\;r_{ij}<D\\ \frac{r_{ij}}{r_{ij}^{3}},\;r_{ij}\geq D \end{array}\right.,$$

where *D* is the damping distance. This approach allows one to prevent the overlapping of oppositely charged species while keeping the exact form of the Coulomb potential at distances larger than *D*; the parameter *D* is essentially the effective bead size and the electrostatic interactions at smaller distances are not important for the system behavior. We used *D* = 0.65 which was show in Reference [28] to be a good choice for the number density of 3, which was used in our work.

The melts (no solvent was added) of diblock-copolymers with one charged block with the length of *N* = 24 were considered; each monomer unit of the charged block carried a charge of +*e*, while the counterions had a charge of -*e* (i.e., the number of counterions was equal to the number of charged monomer units). Since in the classical DPD all the beads have the same size, this is a model of a diblock-copolymer with one block being poly(ionic liquid) with a large free ion. The volume fraction of the uncharged block in the system φ was varied from 5/19 to 11/13; the volume fraction was calculated as $\varphi =\frac{N_{unch}}{N+n}$, where $N_{unch}$ is the length of the uncharged block (which was varied from 10 to 22) and *n* is the number of counterions in the system equal to the number of monomer units in the charged block *n* = *N*-*N_unch_*. If not mentioned, all the Flory–Huggins interaction parameters in the system were equal to 0 and the simulation box size was taken to be 40^3^. The strength of the electrostatic interactions was characterized by a dimensionless parameter $\;\lambda =\frac{l_{b}}{R_{c}}$, where *l_b_* is the Bjerrum length in the system; it was varied from 1 to 20. If we consider parameterization similar to that proposed by Groot [29] with *R_c_* ≈ 0.7 nm, then λ = 1 would correspond to a polar medium with ε≈80 at room temperature, while λ = 20 – to a medium with ε ≈ 4 at room temperature.

The dependence on the position of the order–disorder transition on the chain length was additionally studied for the chains in which the counterions were grafted to the corresponding co-ions of the charged block; all other parameters were the same as for the system above.

Finally, the behavior of copolymers with one charged block was compared to that of corresponding uncharged diblock-copolymers, which were constructed by grafting the counterions to the corresponding co-ions of the charged block with further switching off the electrostatic interactions The incompatibility between the beads constituting different blocks was expressed in terms of the Flory–Huggins parameter χ. Using the approach described in Reference [27], we found that the relation between the value Δ*a* = *a_αβ_ – a_αα_* (*a_αβ_* is the interaction parameter between particles of the types *α* and *β, α* ≠ *β)* and χ for the used parameters (interaction parameter *a_αα_* = *50*, bond stiffness *K* = *4.0*) in the case of homopolymers has the following form: χ*_αβ_* = 0.253 ± 0.004Δ*a_αβ_*; this expression was used in what follows.

The box size in our simulations was equal to 40^3^. Additionally, some systems were studied in simulation boxes with the size of 32^3^ and 48^3^ in order to confirm that the observed structures are in equilibrium.

## 3. Results and Discussions

**Figure 1:**
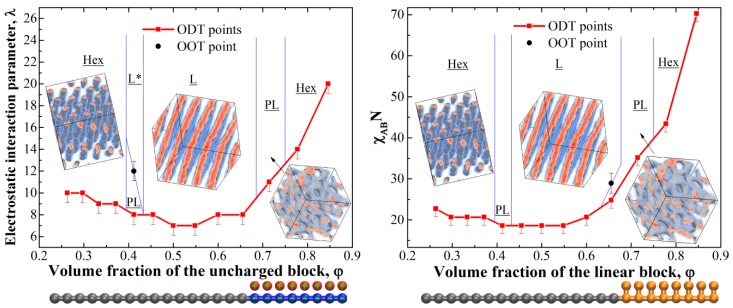
Phase diagrams for melts of diblock-copolymers with one charged block (**left**) and corresponding uncharged diblock-copolymers for the main (linear) chain length of *N* = 24 (**right**); L—lamellae, L*—defective lamellae, PL—hexagonally perforated lamellae, Hex—hexagonally packed cylinders. The lines show the expected positions of the boundaries between the phases and are given as a guide for the eye. The studied chain architectures at φ = 0.5 are shown below each diagram.

First of all, we studied the phase diagram for the diblock-copolymer chains with one charged block. The resulting phase diagram is presented in Figure 1, left; it was calculated in the coordinates (φ – λ), where φ is the volume fraction of the uncharged block in the system.

The diagram is rather similar to the conventional phase diagrams of uncharged diblock copolymers [4,8,10] in terms of the observed phases and their order; it is pronouncedly asymmetric, however, due to the different architecture of the blocks. The uncharged domains are formed by linear blocks, whereas in the domains containing charges, the counterions effectively increase the volume of each monomer unit. The phase diagram was obtained at χ = 0 for all interactions, i.e., the observed microphase separation occurs solely due to the charge-correlation attraction [30]; such attraction is believed to be the reason for the collapse of a single polyelectrolyte chain on non-polar solvent [31,32,33], where similar dense phase consisting of counterions and charged monomer units is observed. The mechanism of the microphase separation seems to be the following: At low values of λ, the electrostatic interactions are weak compared to the energy of thermal motion of counterions, and they try to occupy the maximum volume of the system to increase their entropy and, therefore, the spatially homogeneous phase is most preferable since it corresponds to the maximum volume for the counterions. In this case, there is a strong violation of the local electroneutrality since the charged blocks are “naked” and repel each other. Upon increasing λ, the free energy loss due to the non-electroneutrality becomes higher, and the counterions start to localize near the charges on the blocks, and the correlation attraction [31,34,35] between the blocks causes the formation of ionic domains.

Let us now compare the obtained phase diagram to the phase diagram of the corresponding neutral diblock-copolymer where the behavior is governed by the χ-parameter. In some sense, the polyelectrolyte block and its counterions can be considered as a linear block with one additional bead attached to each monomer unit (such chain architecture is depicted in Figure 1). The phase diagram was calculated in the coordinates (φ – χ_AB_*N*), where χ_AB_ is the Flory–Huggins parameter between the beads of different blocks, which was varied from 0.5 to 2.7, and *N* is the length of the main (linear) chain (i.e., linear block plus linear part of the block with additional beads); it is presented in Figure 1, right. Surprisingly, the diagrams for the charged (Figure 1, left) and the corresponding neutral (Figure 1, right) copolymers are almost identical in those coordinates. The observed differences are rather subtle and can be attributed to the discrete variation of the interaction parameters (λ and χ_AB_) and the subsequent error in the order–disorder transition (ODT) position as well as the presence of the counterion entropy in the charged diblock-copolymer melt. The only marked difference is the presence of the order–order transition (OOT) from PL to L at φ = 0.41 observed in the charged system and absent in the uncharged one. The observed lamellar structures in the charged diblock-copolymers at φ = 0.41 and high λ were prone to the formation of large defects, which might indicate the close proximity of the PL/L transition or even the coexistence of the PL and L phases. The possible reason for such a transition will be discussed below. The dimensionless parameters λ and χ_AB_ seem to play the same role in the microphase separation phenomenon, which is logical: They are both inversely proportional to *k_b_T*. Therefore, the driving force for the microphase separation in the studied diblock-copolymers with one charged block is completely different from that in “classical” diblock-copolymers, but the phase behavior seems to be almost independent of the exact nature of it.

**Figure 2:**
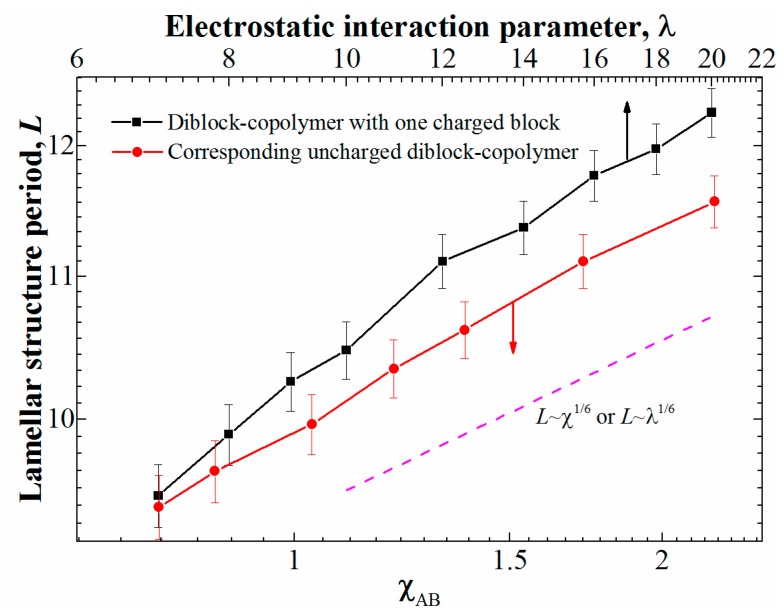
The period of the lamellar structure for φ = 0.5 depending on the interaction parameter: λ for the diblock-copolymers with a charged block and χ_AB_ for the corresponding neutral diblock-copolymers. The ranges of the X-axes are chosen so that they show the same relative increase of the interaction parameter.

However, are the two systems being compared, polyelectrolyte and neutral, are equivalent when it comes to microphase separation? In order to test that, we calculated the dependence of the structure period on the interaction parameters. To that end, we considered the system with φ = 0.5 (i.e., the lamellar-forming copolymer); the box size was increased to 80^3^ to reduce the effect of the finite system size. In order to determine the period of the lamellar structure, we calculated the static structure factor using the standard approach [10]; the position of the first peak gives us the structure period. The obtained dependences are presented in Figure 2.

We can see that for the uncharged copolymer, even though one of its blocks has additional beads attached to one of the blocks, the theoretical dependence for “classical” diblock-copolymers in the strong segregation regime [3] of *L*~χ^1/6^ (with *L* being the structure period) is reproduced. The same scaling *L*~λ^1/6^ is observed for the diblock-copolymer with one charged block at λ≳12; however, at lower values of λ, closer to the transition point, the changes in the lamellar period seem to have a stronger dependence on λ. While the slope difference is rather subtle and can be attributed to the error in the calculation of the structure period, there can be another explanation, namely, the effect of to the counterion entropy: while the system behavior at large λ is dominated by the electrostatic energy (it is much larger than *k_b_T* at the length scales of one monomer unit), at smaller λ the counterion entropy plays an important role and prevents the formation of ordered structures.

**Figure 3:**
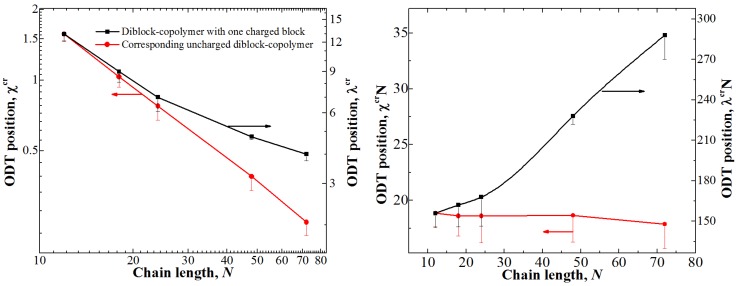
Positions of the ODT for diblock-copolymers with one charged block and corresponding uncharged diblock-copolymer at φ = 0.5 depending on the chain length. The left graph shows λ^cr^ (for diblock-copolymers with one charged block) and $\chi_{\mathrm{AB}}^{\mathrm{cr}}$ (for corresponding uncharged diblock-copolymers), while the right shows the products λ^cr^*N* and $\;\chi_{\mathrm{AB}}^{\mathrm{cr}}N$. The ranges of the Y-axes are chosen so that they show the same relative increase of the interaction parameter.

In order to test this hypothesis, we studied the position of the ODT point for different chain lengths for both types of block-copolymers at φ = 0.5; the obtained dependencies are shown in Figure 3.

The dependence of $\;\chi_{\mathrm{AB}\;}^{\mathrm{cr}}$ on *N* for the uncharged diblock-copolymer is linear (hence, the product $\chi_{\mathrm{AB}}^{\mathrm{cr}}N$ is roughly constant), as it was expected; while the presence of fluctuations leads to a shift in the position ODT point depending on the chain length [36], the studied range of the chain length is small enough so that the differences are within the error. A very different behavior is observed for the diblock-copolymer with one charged block: The product λ^cr^*N* significantly increases with the increase in N. The counterion entropy significantly influences the ODT for longer chains since the transition point for them shifts towards smaller values of λ. We therefore can speculate that in the limit of very long chains the transition point λ^cr^ would approach some finite value of λ upon increasing *N* since the counterion entropy dominates the system behavior at small values of λ.

Another way to show the influence of the counterion entropy is to remove it from the system altogether and investigate the differences. To that end, we studied the position of the ODT point for diblock-copolymers with one charged block in which the counterions were cross-linked to the charged monomer units (i.e., forming dipoles). While the structure of the ionic domain could be somewhat different from that observed in the system with free counterions, the general physical reasons behind the microphase separation are the same. The case with φ = 0.5 was investigated; we found that for the chain length of *N* = 24 the position of the ODT shifted from λ^cr^ = 7 ± 1 to 5 ± 1, i.e., the value becomes ~1.4 times smaller, while for the chains with *N* = 72 the difference is much more pronounced: From λ^cr^ = 4 ± 0.5 to 1.6 ± 0.2, i.e., the value becomes ~2.5 times smaller. These data support our hypothesis about the influence of the counterion entropy once again: It is more significant when the microphase separation is observed at small λ, i.e., for long chains.

It is worth noting that since the microphase separation occurs due to the effective attraction (in our case it is the charge-correlation attraction), the domains formed by the attracting beads (i.e., ionic domains) in DPD will inevitably have larger density compared to the other domains (i.e., neutral domains). For the chosen parameters, at large values of λ > 14 at φ = 0.5 we obtained that the ionic domains have ~6–7% higher number density compared the average system value of ρ = 3. The deviations can be made smaller (but not completely removed) by increasing the repulsion parameter between alike species *a_αα_* and/or the electrostatic damping distance *D*. However, the former greatly increases the probability of the system being caught in a kinetically trapped state even at low segregation strengths, while significant changes in the latter will decrease the accuracy of taking into account the electrostatic interactions [28]. We additionally tested a system at φ = 0.5 with *a_αα_* = 150 at λ = 16. The obtained defective lamellar structure had the same period (defined by the position of the first peak on the static structure factor) as that depicted in Figure 2 within the calculation error. The changes in the domain densities obviously give rise to a slight shift of the effective volume fraction of the components; we suppose that this is the reason for the observed transition PL → L transition observed at φ = 0.41 for the diblock-copolymers with one charged block. Increasing λ caused a minor decrease in the effective volume fraction of the charged block, which was enough for the system to undergo the transition due to the proximity of the phase boundary.

**Figure 4:**
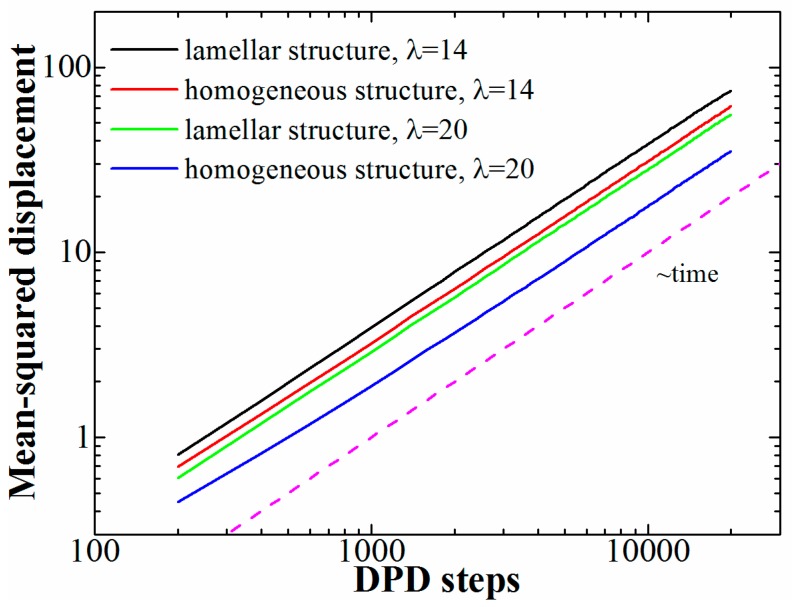
Mean-squared displacement of counterions at different values of λ (14 and 20) obtained for lamellar structures (diblock-copolymers) and homogeneous structures (copolymers with evenly distributed charged groups). The diffusion coefficient is reflected by the position of the curve: The higher the curve, the higher the diffusion coefficient.

As was mentioned in the introduction, copolymers with polyelectrolyte blocks are often considered as new-generation materials for ion-exchanging membranes. For such applications, the ion conductivity is a crucial characteristic. Ion conductivity is related to the diffusion of free ions; therefore, we studied the influence of the presence of ordered structures on the ion diffusion. To that end, we compared the mean-squared displacement (MSD) of the counterions in lamellar structures at φ = 0.6 to that in melts of copolymers in which the charged groups were distributed evenly, but their number as well as the chain length was the same. The latter copolymers did not form any ordered structures even at the highest studied λ; the results are presented in Figure 4. The mean-squared displacement for the lamellar structures was calculated along one axis in the lamellar plane (the diffusion perpendicular to the lamellae is limited by the domain size); for the copolymers with evenly distributed charged groups the diffusion is isotropic, so any direction can be chosen.

First of all, we can see that in all studied cases the MSD shows a linear relationship to time, which means that the counterions undergo normal diffusion. No stable ion pairs are formed, and the counterions are “shared” between all the charged monomers (despite rather high values of λ) like electrons in metals. Similar behavior was observed in globules formed by a single polyelectrolyte chain [33,37], in which the reason for the formation of the collapsed state is also the charge-correlation attraction. Second, we see that for both studied values of λ the diffusion in the ordered structures is faster; the difference is more pronounced at the higher λ-value.

Due to the homogeneous structure of the melts of copolymers with evenly spaced charged groups, the movement of the counterions is slowed down by the presence of neutral regions in the system; in some sense, there is no direct pathway formed by charged monomer units for a counterion between two points at any given moment of time. At large values of λ the charged groups seem to form small clusters surrounded by the neutral monomer units, which restricts the movement of the counterions. This can be easily understood if one considers a copolymer with only a small fraction of charged groups; if these groups are distributed evenly (or randomly) and the system are homogeneous, the charged groups are essentially isolated from one another and the counterions form stable ion pairs with the monomer units. The diffusion is possible through the segmental movement and “hopping” of counterions between the ion pairs, but is it much slower than the diffusion in a microphase-separated state if all the charged groups form one block; in that case, the counterions can freely move within the domains formed by the ionic species.

## 4. Conclusions

In this work we investigated the phase behavior of melts of block-copolymers with one charged block by means of dissipative particle dynamics with explicit electrostatic interactions; such systems are a model of, for example, diblock-copolymers with one block being ionic liquid. We assumed that the blocks are fully compatible, i.e., all the Flory–Huggins χ parameters were equal to 0. This way, we showed that the charge correlation attraction solely can cause microphase separation with long-range order; a phase diagram was constructed by varying the volume fraction of the uncharged block and the electrostatic interaction parameter λ. Conventional phases like lamellae, hexagonally perforated lamellae, and hexagonally packed cylinders were observed. The obtained phase diagram was compared to the phase diagram of corresponding neutral diblock-copolymers with non-zero χ-parameter between the beads of different blocks; one block of such copolymers consisted of “dumb-bell” monomer units to mimic the increase of the volume of each monomer due to the presence of counterions. Surprisingly, the differences between these phase diagrams are rather subtle; the same phases in the same order are observed, and the positions of the ODT points are similar if the λ-parameter is considered as an “effective” χ parameter. Therefore, the dimensionless parameters λ and χ seem to play the same role in the microphase separation phenomenon; the phase behavior seems to be almost independent of the exact nature of the driving force behind it.

Next, we investigated how the presence of free counterions and their entropy affects the behavior of the system. To that end, we studied the position of the ODT for a lamellar structure depending on the chain length *N*. It turned out that while for the uncharged diblock-copolymer the product $\chi_{\mathrm{AB}}^{\mathrm{cr}}N$ was almost independent of *N*, which is in line with the theoretical predictions for “classical” diblock-copolymers with symmetric blocks, for the diblock-copolymers with one charged block we observed a significant increase in λ^cr^*N* upon increasing *N*. We suppose this can be attributed to the fact that the counterion entropy prevents the formation of ordered structures, and its influence is more pronounced for longer chains since they undergo the transition to ordered structures at smaller values of λ, when the electrostatic energy becomes comparable to *k_b_T*. This was supported by studying the ODT in diblock-copolymers with charged blocks and counterions cross-linked to the charged monomer units (thus forming dipole monomers). Indeed, the ODT for such systems was observed at significantly lower values of λ with the difference being more pronounced at longer chain lengths *N*. Therefore, we can speculate that in the limit of  N→∞ there will be a finite value of λ necessary to observe the ODT in melts of diblock-copolymers with one charged block and free counterions unlike uncharged diblock-copolymers where $\chi^{\mathrm{cr}}\to 0\;$ when  N→∞.

Finally, we studied the diffusion of counterions in the obtained ordered structures and compared it to the case of a system with the same number of charged groups but a homogeneous structure; the diffusion coefficient along the lamellar plane was found to be higher than in any direction in homogeneous structure. This can be attributed to the fact that in homogeneous structures the movement of the counterions is restricted by the presence of uncharged monomers surrounding the charged ones, while the formation of purely ionic phases in the case of ordered structures allow the counterions to move freely within the domains.

It is worth noting that in this work (as well as in previous work [22] on the subject) the size of the monomer units and counterions is the same. However, the difference in the ion sizes can have a dramatic effect on the formation of dense ionic structures; for example, recent studies on the single polyelectrolyte chain collapse [33,37] have shown that the steric restrictions due to the ion size mismatch can significantly shift the position of the collapse. It seems that similar reasoning can be applied to the correlation attraction-driven microphase separation, and the latter is influenced by the ratio of the ion sizes. Effect of the ratio of the ions’ sizes on the system behavior could be an interesting topic for a future study.

In summary, in this work, we shed some light on the complex and intriguing problem of self-organization of copolymers with charged units. The fact that the microphase separation is observed even at zero Flory–Huggins parameter can be used for the creation of “high-χ” copolymers: The incorporation of charged groups (for example, ionic liquids) can significantly increase the segregation strength.
